# A Card

**Published:** 1889-09

**Authors:** Jos. Jacobs


### EDITORIALS AND MISCELLANEOUS.

THE PHARMACEUTICAL ASSOCIATION OF GEORGIA expels a member, whom it seems was not a member as the following card from the aggrieved party explains:

A CARD.

At a meeting of the Georgia Pharmaceutical Association, held at Salt Spring, near Atlanta, Georgia, during the month of July, 1889, the undersigned was expelled from that association, the pretence being, that I had been guilty of selling adulterated drugs! As I was not present, nor did I even have notice of the fact, that charges had been or would be preferred against me, I cannot of course say how the Association arrived at the conclusion that I ought to be expelled. My first and only knowledge of the whole transaction was obtained from the news papers which stated briefly that I had been expelled for "adulterating drugs." As will be seen by reference to the printed Journal of the annual proceedings of this Association for the years 1886, 1887 and 1888, my name does not appear as a member, in the list of members of said Association therein published. These proceedings can be had of the Sect'y. of the Association, Mr. Slack, of LaGrange, Georgia. To those at all familiar with the circumstances leading up to this final act of the Association and of the members who so craftily engineered it, is unnecessary for me to explain, that unsuccessful competition, envy and malice, are and were the sole reasons for this outrageous action. As I do not propose to submit to this unheard of proceeding on the part of this fair minded (?) Association which arrogates to itself the right to try and expel one, whom they refuse to recognize as a member, and that too, without notice, or a hearing. I will not go into the question further at this time, but will await the proceeding, which my attorneys, Messrs. Weil & Brandt, of Atlanta, Georgia, will institute for me, in the courts, where the animus and the truth of the alleged charges on which I was tried unknown to me, by this Association, can be legally investigated.

JOS. JACOBS.

